# Isolation and characterization of canine adenovirus type 2 (CAV-HN45) and its selective infection of human cervical cancer cells with preliminary oncolytic potential

**DOI:** 10.3389/fvets.2025.1692395

**Published:** 2025-10-28

**Authors:** Cong-Rong Wang, Dong-Mei Wang, Guo-You Yin, Yan-Hong Wang, Min Lu, Zhuo-Wei Zhang, Yue Wen, Ding-Zhuo Gao, Jun Hong, Peng-Fei Fu

**Affiliations:** ^1^College of Life Science and Engineering, Henan University of Urban Construction, Pingdingshan, Henan, China; ^2^College of Veterinary Medicine, Henan Agricultural University, Zhengzhou, Henan, China

**Keywords:** canine adenovirus 2, isolation, characterization, phylogeny, infection spectrum, oncolytic potential

## Abstract

Canine adenovirus type 2 (CAdV-2) infects the respiratory tissues of dogs and induces canine infectious laryngotracheitis. CAdV-2 has a high incidence of infection and is easily co-infected with other viruses. Moreover, CAdV-2 is a mammalian adenovirus with characteristics similar to those of Human Adenovirus Type 5 (HAdV-5), making it a promising candidate for recombinant vaccine development and gene therapy applications. In this study, we isolated and identified a CAdV-2 strain (CAV-HN45) and investigated its growth characteristics and viral tropism by evaluating its infection efficiency in various cell lines. Our findings demonstrate that CAV-HN45 can effectively infect cells of swine, canine, and human origin. *In vitro*, CAV-HN45 efficiently infected HeLa cells and showed selective infectivity toward human cervical cancer cells, although replication capacity declined after serial passages. This study provides a reference for the future studies on adenovirus vaccine vectors with high selective expression, potentially offering promising applications in the treatment of human cancers.

## 1 Introduction

Canine Adenovirus (CAdV) is one of the most pathogenic and widely distributed viruses within the mammalian adenovirus family (1). Canine Adenovirus Type 1 (CAdV-1) was first identified in 1925, primarily causing infectious hepatitis in dogs, as well as in foxes and bears (2). Canine Adenovirus Type 2 (CAdV-2) was initially isolated from dogs with laryngotracheitis in 1961 and was named Toronto A26/61 (3). CAdV-2 can proliferate within the canine digestive tract, leading to the development of canine diarrhea. CAdV-2 is a mammalian adenovirus that causes laryngotracheitis and diarrhea in dogs (4–6).

CAdV-2 is a non-enveloped, icosahedral virus with a diameter ranging from 70 to 100 nm. It is coated with self-encoded capsid proteins, mainly composed of fiber, penton base, and hexon. The hexon carries genus- and subgenus-specific antigenic determinants, playing a crucial role in distinguishing different virus strains. The fiber is pivotal in CAdV-2 infection by facilitating fusion between the virus and host cell receptors (7). The genome of CAdV-2 is a linear, non-segmented, double-stranded DNA (1, 8). Among the early transcription units of CAdV-2, the E3 gene predominantly encodes non-essential proteins. E3 is associated with impairing host defense functions through its encoded proteins, which possess immunosuppressive effects.

Vectors based on human adenovirus type 5 (HAdV-5) have been extensively investigated. Although the HAdV-5 vector primarily targets cells with high chimeric antigen receptor (CAR) expression, it encounters challenges in targeting cells with marginal CAR expression, resulting in viral toxicity. Currently, there is a growing interest in identifying alternative mammalian adenovirus vectors that can replace human adenovirus vectors to achieve improved immunotherapy outcomes. Studies have demonstrated that CAdV-2 can effectively infect HAdV-5-uninfected cells, and its infection is independent of CAR expression (9). The tropism of CAdV-2 differs from that of HAdV-5, and CAdV-2 vectors can transduce HAdV-5-refractory cells, such as neurons and ovarian cancer cells (10, 11). Moreover, the use of CAdV-2 vectors can overcome interference from maternal antibodies on immunotherapy efficacy. Considering these distinctive characteristics, the CAdV-2 vector represents a promising candidate for replacing human adenovirus vectors.

In this study, we characterized a canine-derived CAdV-2 isolate named CAV-HN45 to investigate its infection tropism across various cell lines *in vitro*, providing valuable insights for developing novel gene delivery tools and advancing research on CAdV-2 vectors.

## 2 Materials and methods

### 2.1 Sample collection

A rectal swab was collected from a Golden Retriever exhibiting symptoms of diarrhea and vomiting on August 23, 2020, in Anyang City, Henan Province of China. The clinical sample was mixed in 1.0 ml of phosphate-buffered saline solution (pH 7.2). Aliquots of 200 μl from the above mixture of sample were taken for detecting DNA/RNA viruses. All samples were stored at −80 °C to prevent virus deactivation.

### 2.2 Virus isolation

The clinical samples were homogenized in DMEM containing 100 IU/ml penicillin and 100 μg/ml streptomycin and centrifuged at 5,000 rpm for 3 min at 4 °C. Supernatants were filtered through 0.22 μm filters (EMD Millipore, Billerica, MA, USA) and inoculated into MDCK cells were incubated at 37 °C for 1 h. After viral adsorption, the inoculum was removed, and cells were overlaid with 1.5% low melting gel (Solarbio, Beijing, China) in DMEM containing 2% FBS (12, 13). The plates were incubated at 37 °C with 5% CO_2_ until plaques became visible. Isolate cytopathic effects (CPEs) plaques and further inoculate onto MDCK cells, viruses were harvested by three freeze–thaw cycles, stored at −80 °C.

### 2.3 Virus titer assay

MDCK cells infected with virus isolated and identified were harvested and observed when CPE lesions were complete under a microscope, and the virus titer was evaluated via a 50% tissue culture infective dose (TCID_50_) assay. The MDCK cells were inoculated into 96-well plates and incubated in DMEM containing 5% NBS. The virus samples were 10-fold serially diluted with DMEM, inoculated into MDCK cells, and cultured at 37 °C for 48–72 h. CPE was observed after MDCK cell infection by the virus, and TCID_50_ was calculated using the Reed–Muench method.

### 2.4 Identification of virus isolate

Identification of virus isolate involved PCR assay, Electron microscopy and western blotting techniques. DNA extraction of viruses was performed with the viral DNA/RNA extraction kit (TIANGEN, China). Extracted nucleic acids were subjected for PCR amplification primers HA1 and HA2 (Table 1) for detection of CAdV-1/2 (14). Using previously published primers and methods suitable for both CAdV-1 and CAdV-2 were used for the PCR (14). Consequently, the formation of PCR products in the expected sizes (508 and 1,030 bp for CAdV-1 and CAdV-2, respectively) was analyzed using DNA gel electrophoresis.

**Table 1:** Sequences of the primers utilized in this study.

**Primers**	**Sequences (5^′^-3^′^)**
HA1	5′-CGCGCTGAACATTACTACCTTGTC-3′
HA2	5′-CCTAGAGCACTTCGTGTCCGCTT-3′
Penton Base-F	5′-ATGGAGTTTTCGTCGTCTCCTC-3′
Penton Base-R	5′-CTAGAAGGTTTTACTGGACAGC-3′
Fiber-F	5′-TTGGTACTTCCACTTGTGCG-3′
Fiber-R	5′-TAACTTTTCCTGAAGGCGGC-3′
Hexon-F	5′-TGAGAAGATGGCAACCCCGTCGATG-3′
Hexon-R	5′-TATTAGCTTAGGTGGTGGCGTTGCC-3′

Virus particles were purified from cell culture supernatants by ultracentrifugation at 35,000 rpm for 2 h and resuspended in PBS. For negative staining, 5 μl of the viral suspension was applied to a carbon-coated copper grid, stained with 2% uranyl acetate, and air-dried. Grids were imaged using the FEI Tecnai G2 F20 operated at 120 kV. Transmission electron microscopy was performed. Images were analyzed using ImageJ software to measure the size distribution of viral particles.

The viral isolate was further characterized by indirect western blotting techniques using a monoclonal antibody against the CAdV penton base protein (prepared and stored by our laboratory). Virus-infected cells were lysed in radio immunoprecipitation assay buffer, and protein concentrations were determined using a bicinchoninic acid assay (Ding Guo, Beijing, China). Equal amounts of protein (20 μg per lane) were separated by 10% SDS-PAGE and transferred to a polyvinylidene fluoride membrane (Millipore, USA). The membrane was blocked with 5% non-fat milk (Sangon, China) in TBST and incubated with a primary antibody against the CAdV penton base protein (1:1,000 dilution) or anti-β-actin antibody (1:5,000 dilution, Sigma-Aldrich, USA) overnight at 4 °C. After washing, the membrane was incubated with an HRP-conjugated secondary antibody (1:5,000 dilution, Jackson ImmunoResearch Laboratories, Inc., USA) for 1 h at room temperature. Immunoblotting results were visualized with Luminata Crescendo Western HRP Substrate (Millipore, USA) on a GE AI600 imaging system.

### 2.5 Sequencing and phylogenetic analysis

According to the analysis of the constant regions of CAdV-2 fiber, penton base, hexon, and E3 sequences published by NCBI, specific primers for PCR amplification were designed (Table 1). All the purified PCR products were cloned into the pMD19-T vector (TaKaRa, Beijing, China) for sequencing (Sangon, Shanghai, China). The contig assemblies of fiber, hexon, penton base, and E3 of CAV-HN45 were arranged using the DNASTAR 7.0 software (DNASTAR, Inc., USA) and were compared with those of reference CAdV strains obtained from the National Center for Biotechnology Information (NCBI) nucleotide database. All details concerning the virus isolate sequences in this study were submitted to GenBank. E3 sequences were aligned at the nucleotide level by Mega 7, whereas fiber, penton base and hexon sequences were aligned at the amino acid level by Mega 7 and DNASTAR 7.0 and manually adjusted. The nucleotide sequence and amino acid sequence of the isolated and reference strains were analyzed. A phylogenic tree was constructed with the neighbor-joining method and 1,000 bootstrap replicates.

### 2.6 Immunofluorescence assay (IFA) for cell line susceptibility

Different cells infected with CAdV-2 in 24-well plates were washed twice with PBS and fixed in 4% paraformaldehyde in PBS and then permeabilized with 0.1% Triton X-100 in PBS. Cells were then incubated with the mouse anti-CAdV-2 at 1:1,000 dilution for 1 h at 37 °C, washed with PBS, and stained with the Alexa Fluor 488-conjugated goat anti-mouse secondary antibody (Thermo Fisher Scientific, USA) at 1:1,000 dilution. After incubation for 1 h at 37 °C, the cells were washed with PBS, stained with 4′,6-diamidino-2-phenylindole (DAPI) at 1:1,000 dilution, and visualized on a fluorescence microscope.

## 3 Results and discussion

CAdV-2, a double-stranded DNA virus, has been reported to cause severe necrotizing bronchitis and interstitial pneumonia in the presence of other pathogens or an immune-compromised host (15). Additionally, it is associated with fatal cases of diarrhea and neurological disease in dogs (6, 16, 17). This study confirms CAdV-2 infection in a dog through PCR assay. Swab samples were extracted for genomic DNA/RNA testing of common canine viruses by PCR/RT-PCR, resulting in positive detection for CAdV-2 while CDV, CPV, CAdV-1, and CCV were all negative.

**Figure 1:**
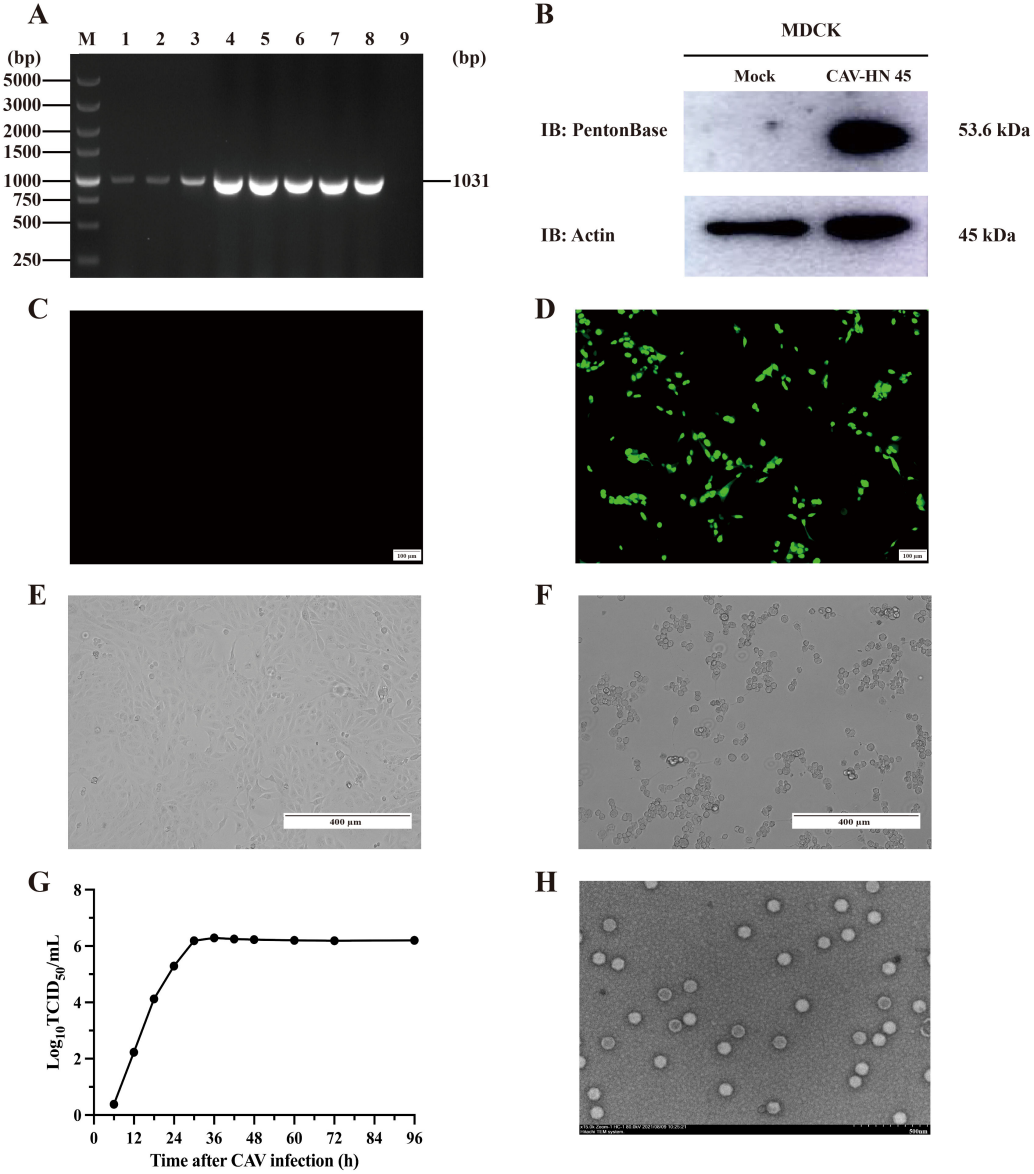
Isolation and identification of CAdV-2 strain CAV-HN45 in MDCK. **(A)** Identification of CAV-HN45 cultured in MDCK cells at serial passages, by PCR. M: DNA marker, 1: Sample, 2–8: Sample of P1-P7 virus generation, 9: Negative sample. **(B)** MDCK cells were either mock (uninfected) or infected with CAV-HN45 for 24 h and then lysed for immunoblotting analysis with the viral protein Penton base. β-Actin was used as the loading control. Detection of CAV-HN45 by immunofluorescence assay at **(C)** control (uninfected) MDCK cells and **(D)** CAV-HN45 infected MDCK cells. Virus replication and cytopathic effect (CPE) in MDCK cells infected with CAV-HN45. Cells were left uninfected **(E)** or were infected with CAV-HN45 **(F)** and imaged using brightfield microscopy. **(G)** One-step growth curves of CAV-HN45 (MOI = 0.1) in MDCK cells. This experiment was performed three times, and the results are shown as the mean ± SD. **(H)** Electron micrograph of purified CAV-HN45 particles after negative staining.

The MDCK cell line was utilized for the isolation and purification of CAdV-2 from positive swab samples. The virus was plaque-purified in MDCK cells and subsequently passaged through seven generations. The isolate was obtained via plaque purification, and the virus strains derived from culture were subjected to PCR assay, Western blotting, and IFA. PCR targeting the E3 gene of CAdV-2 from swab samples and cell culture supernatants containing CAdV-2 revealed DNA products of the expected size (1,030 bp; Figure 1A). These results confirm the presence of CAdV-2 nucleic acid material in the isolated virus. Western blotting, using an anti-CAdV penton base mouse monoclonal antibody as the primary antibody, showed a target band at 50–70 kDa (Figure 1B). The presence of CAdV-2 in the isolate was further confirmed by IFA (Figures 1C, D). Mouse anti-CAdV fiber protein monoclonal antibodies were used as primary antibodies, and Alexa Fluor 488-conjugated goat anti-mouse antibodies were employed as secondary antibodies to detect CAdV-2. Specific green fluorescence signals were observed in the CAV-HN45 isolate, but not in control cells. Overall, these results confirm the presence of CAdV-2 in our isolated sample, which we have designated as “CAV-HN45.”

The isolated and purified virus was subjected to analysis of its biological characteristics. The replication of the virus in MDCK cells was assessed using a single-step growth curve (Figure 1G). As shown in Figure 1G, the one-step growth curve indicated that the CAV-HN45 titer reached 1 × 10^6.23^ TCID_50_/ml at 30 h post-infection (hpi) and remained constant thereafter. CPEs were observed under the microscope as early as 48 hpi, characterized by cell rounding and detachment (Figures 1E, F). Electron microscopic examination of the purified virus preparation revealed typical adenovirus particles. Negative staining observation using an electron microscope showed numerous well-arranged adenovirus-like particles with icosahedral symmetry, measuring approximately 80 nm in diameter (Figure 1H), with no other particles detected. The purified viral titer was determined to be 1 × 10^6.23^ TCID_50_/ml using the Reed–Muench method.

The isolated CAdV-2 was further characterized in terms of its molecular biological characteristics. Given that the pathogenicity and affinity of adenovirus may be closely related to the major structural proteins of the capsid, we conducted cloning, sequencing, comparison, and phylogenetic analysis of its fiber, penton base, hexon, and E3.

The sequences of the fiber, penton base, hexon, and partial E3 of the identified CAdV-2 strains have been deposited in GenBank, with accession numbers shown in Table 2. Sequence analysis, based on both the CAdV-2 strains from this study and the CAdV-2 reference strain (U77082) from GenBank, demonstrated that the isolated strain in this study has three nucleotide mutations (G1473A, T2664G, and G2675T) in the hexon protein sequence, one of which is a non-synonymous mutation (G892V). An amino acid mutation (A471V) was also identified in the penton base protein. In contrast, the fiber and E3 proteins did not exhibit any unique amino acid mutations and remained genetically stable.

**Table 2:** Relative information of the CAV-HN45 in this study.

**Gene**	**Accession number**	**Size CAdV-2 (Nucleotide)**	**Size CAdV-2 (Amino Acid)**
Fiber	OP618113	1,629 bp	57 kDa
Penton Base	OP618114	1,434 bp	53.6 kDa
Hexon	OP618115	2,718 bp	101.2 kDa
E3	OP618116	1,031 bp	-

**Figure 2:**
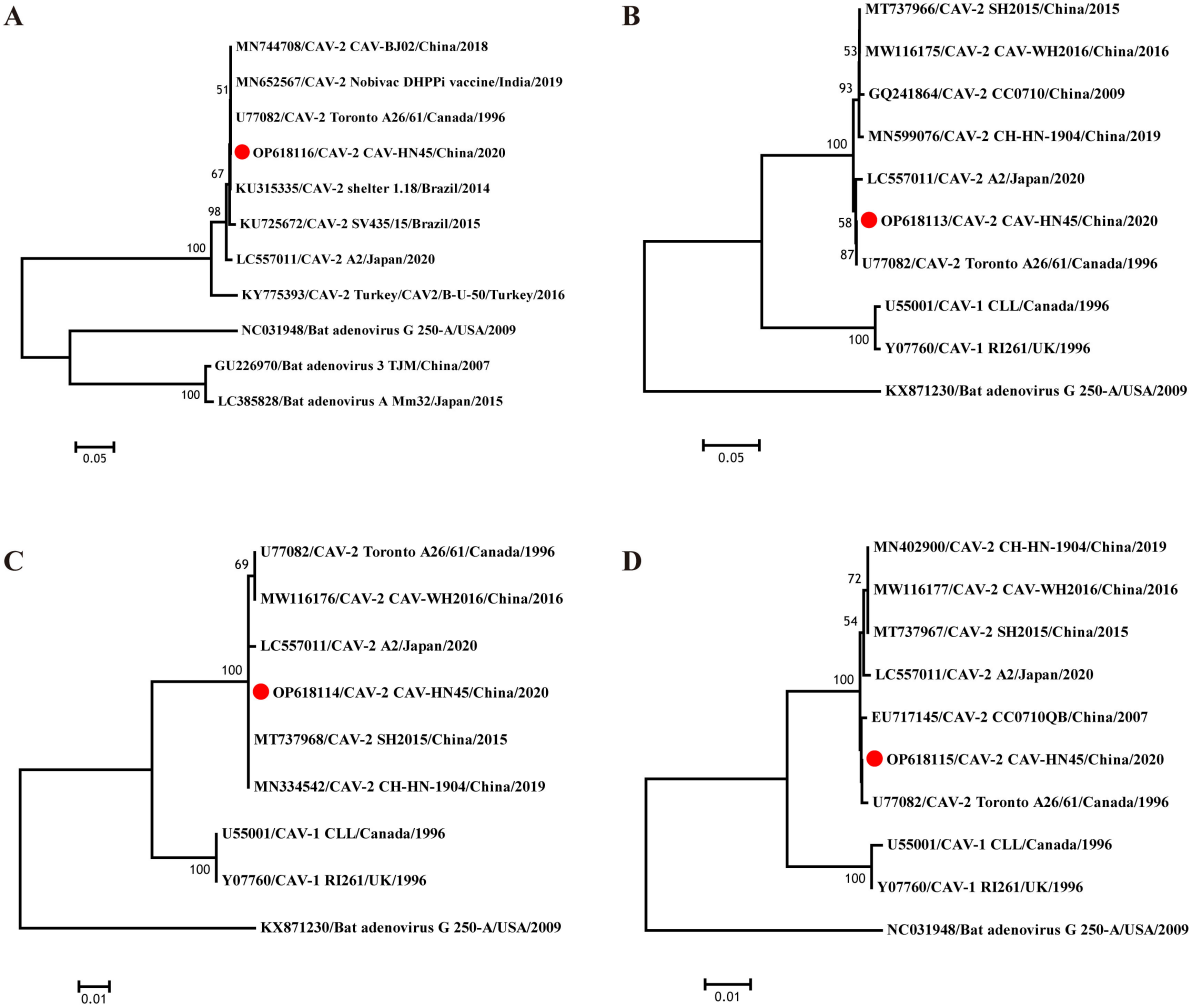
Evolutionary relationships of CAV-HN45. Phylogenetic tree constructed using the complete sequences of **(A)** E3, **(B)** fiber, **(C)** penton base, and **(D)** Hexon of canine and bat adenoviruses. The phylogenetic tree was constructed using nucleotide sequences generated in this study and the sequences of CAdV-1, CAdV-2, and bat adenovirus reference strains obtained from GenBank. Based on the sequences, a phylogenetic tree was constructed using the MEGA 7 software with the neighbor-joining (NJ) method. The reliability of the phylogenetic tree was verified through the bootstrap method with 1,000 replicates. The tree is drawn to scale, with branch lengths in the same units as those of the evolutionary distances used to infer the phylogenetic tree.

Furthermore, the sequences generated in this study were compared with other available CAdV-2 sequences from GenBank to construct a phylogenetic tree and elucidate the genetic heterogeneity among different CAdV-2 strains (Figure 2). The virus identified in this study exhibited high similarity with other isolates from China and vaccine strains, belonging to the type 2 clade and forming part of the subgroup containing the U77082 strain. Specifically, the E3 gene of CAV-HN45 was closely related to the Indian vaccine strain (MN652567) and reference strains from Brazil (KU315335, KU725672). The fiber of CAV-HN45 showed a close relationship with isolates from Shanghai and Japan in recent years but was not in the same branch as some isolates from central China (18). The penton base was most closely related to Chinese isolates (MT737968, MN334542), and the hexon was closely related to the reference strain (U77082).

CAdV infection has a global distribution among various animal groups, including dogs, foxes, bears, wolves, and raccoons (4, 19, 20). Anti-CAdV-2 antibodies have been detected in foxes, bears, and pandas, and CAdV-2 has been identified in the central nervous system (CNS) of dogs and foxes (1, 21, 22). Some studies have suggested that CAdV-2 has not yet crossed the species barrier and cannot replicate in human cells (23).

**Figure 3:**
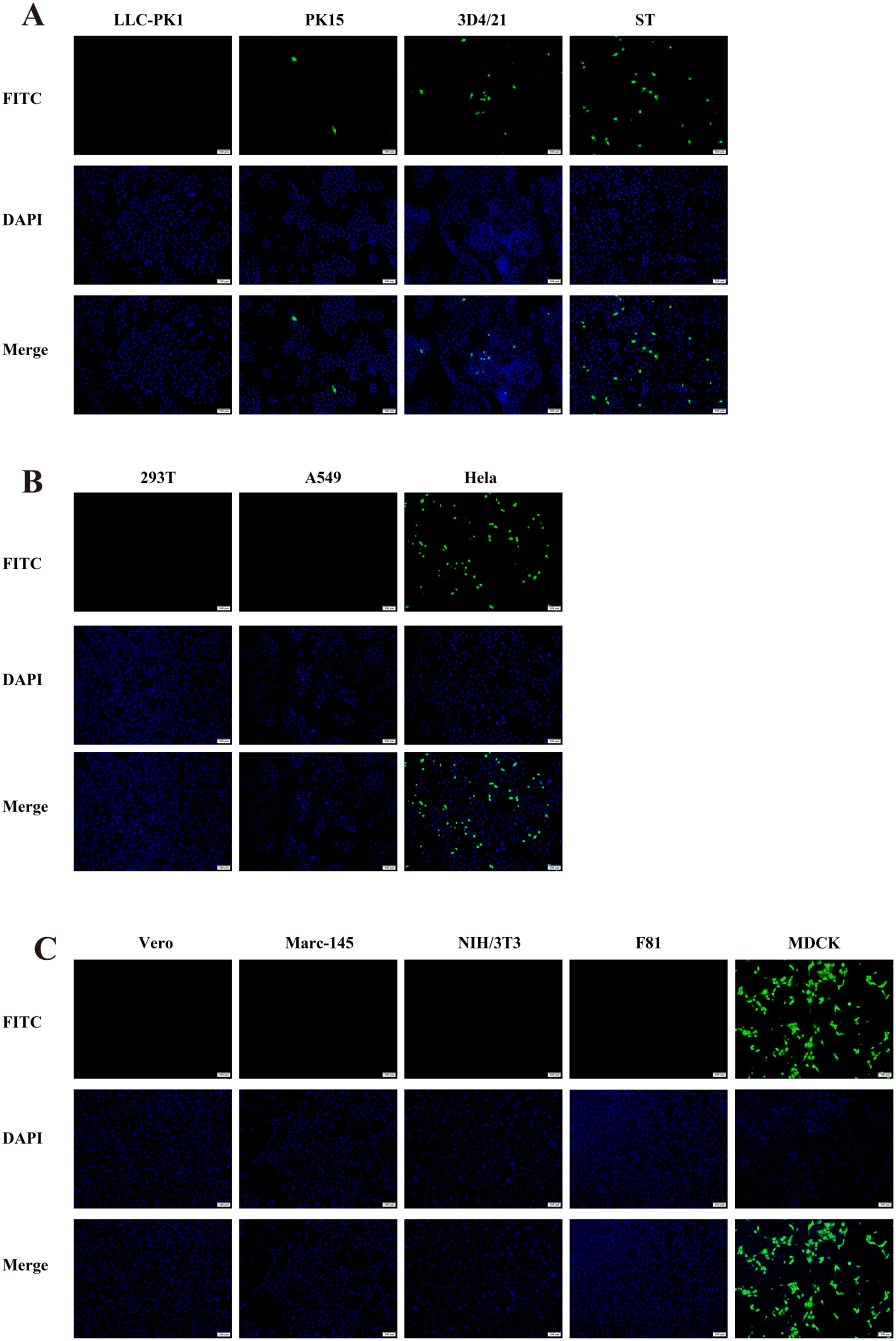
Immunofluorescence assay showing the susceptibility of different cell lines to CAV-HN45 infection. (10 × ). Immunofluorescence assay of cells infected with CAV-HN45 at an MOI of 0.01 was performed using mouse anti-CAV-FIBER monoclonal antibody and Alexa Fluor 488-conjugated anti-rabbit IgG as the secondary antibody, with DAPI for visualization of cell nuclei. Mock-infected cells were treated with the same procedures as appropriate. Cells were tested from different species of origin, including swine (LLC-PK1, PK15, 3D4/21 and ST) **(A)**, human (293T, A549, and HeLa) **(B)**, monkey (Vero and Marc-145), mouse (NIH/3T3), cat (F81) and canine (MDCK) **(C)**.

In this study, we found that the CAV-HN45 isolate is highly infectious to canine kidney cells (MDCK). Additionally, it was able to infect porcine kidney (PK15), lung (3D4/21), and testis (ST) cells, as well as human cervical cancer cells (HeLa). However, CPEs were only observed in MDCK and HeLa cells (Figure 3 and Table 3). This selective CPE manifestation may be related to amino acid mutations in the hexon and penton base proteins (24, 25). But we only obtained partial sequences of the E3, fiber, hexon, and penton base genes. Whether this isolate has deletions or mutations in the E3 gene or other gene regions, and whether these genetic alterations are related to the observed phenomenon, require further investigation.

**Table 3:** Summary of human and animal cell lines and their susceptibility to CAV-HN45 infection as determined by CPE and IFA.

**Cell line information**			**Results of** ***in vitro*** **infection with CAV-HN45**^**a**^	
**Species and/or tissue origin**	**Name**	**ATCC no**.	**IFA**	**CPE**
**Swine**				
Kidney	LLC-PK1	CL-101	–	–
	PK15	CCL-33	+	–
Alveolus	3D4/21	CRL-2845	+	–
Testis	ST	CRL-1746	++	–
**Human**				
Kidney	293T	CRL-3216	–	–
Lung carcinoma	A549	CCL-185EMT	–	–
Cervix adenocarcinoma	HeLa	CCL-2	+++	++++
**Monkey**				
African green monkey kidney	Vero	CRL-1586	–	–
Kidney	Marc-145	N/A	–	–
**Mouse**				
Embryo fibroblasts	NIH/3T3	CRL-1658	–	–
**Cat**				
Kidney	F81	N/A	–	–
**Canine**				
Kidney	MDCK	CCL-34	++++	++++

^a^Degree of infection as determined by IFA or CPE (–, no infection or obvious lesion [ ≤ 1%]; +, ≤ 25%; ++, ≤ 50%; +++, ≤ 75%; ++++, ≤ 100%).

N/A, Not available.

On the other hand, adenoviruses primarily infect epithelial cells, and the majority of human tumors originate from epithelial tissues. Moreover, adenoviruses are among the most promising oncolytic virus (OV) candidates. OV therapy is a promising approach that involves the use of natural or genetically modified viruses to selectively destroy tumor cells and induce anti-tumor immunity (26). Several studies have utilized genetic engineering to develop recombinant HAdV-5 (H101) as an oncolytic adenovirus for the treatment of hepatocellular carcinoma, cervical cancer, liver cancer, and malignant tumors with pleural effusion and ascites (27–29).

**Figure 4:**
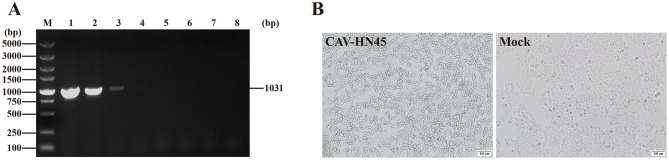
CAV-HN45 infects the HeLa cell line. **(A)** Identification of CAV-HN45 cultured in HeLa cells at serial passages, by PCR. M: DNA marker, 1: positive control, 2–7: sample of P5-P10 virus generation, 8: negative control. **(B)** HeLa Cells were infected with CAV-HN45, mock were normal HeLa cells and imaged using brightfield microscopy (10 × ).

Our results showed that CAV-HN45 replicated in HeLa cells and induced cell death, but its replication capacity was lost after six passages (Figure 4). Sequencing of key viral genes (E3, fiber, hexon, and penton) at passage six revealed no mutations, suggesting that this loss may instead reflect host antiviral responses, defective particle accumulation, or epigenetic regulation; unsequenced loci could also play a role, and broader genomic analyses including full-genome sequencing and reverse genetics may help clarify the underlying cause in future studies.

In this study, CAV-HN45 was able to infect HeLa cells but showed no infectivity toward 293T cells (human embryonic kidney), providing preliminary evidence of selective activity toward cervical cancer cells. Although 293T cells are not fully representative of normal primary cells, future studies with additional non-cancerous human cell lines will help to more rigorously validate this selectivity. Our observation that CAV-HN45 was able to infect HeLa cells complements recent advances using CAdV-2–derived vectors in the veterinary setting, where intratumoral administration has been reported to be safe and immunologically active in canine patients (30). Further investigation is required to determine whether this infectivity is unique to this strain or if other CAdV-2 strains also exhibit infectivity in HeLa cells.

## 4 Conclusions

In summary, we isolated and characterized a CAdV-2 strain (CAV-HN45) from dog secretions. The strain displayed typical adenoviral morphology, stable replication in MDCK cells, and close phylogenetic relatedness to other CAdV-2 isolates in China. Notably, CAV-HN45 was able to infect human cervical cancer cells (HeLa), showing selective tropism not previously reported for canine adenoviruses. Although the mechanisms remain unclear, further mechanistic studies and *in vivo* evaluation are warranted. These findings highlight the potential of CAV-HN45 as a candidate for developing vaccine vectors or recombinant oncolytic viruses with targeted applications in tumor therapy.

## Data Availability

The datasets presented in this study can be found in online repositories. The names of the repository/repositories and accession number(s) can be found below: https://www.ncbi.nlm.nih.gov/nuccore/, OP618113; OP618114; OP618115; OP618116.

## Supplementary material

Supplementary Image 1 β-Actin internal control.

Supplementary Image 2 Penton base protein detection.
