# Supplementary figures and images for: Isolation and characterization of canine adenovirus type 2 (CAV-HN45) and its selective infection of human cervical cancer cells with preliminary oncolytic potential
Source: Front Vet Sci. 2025 Oct 28;12:1692395. doi: 10.3389/fvets.2025.1692395 (PMC12604354; doi:10.3389/fvets.2025.1692395)

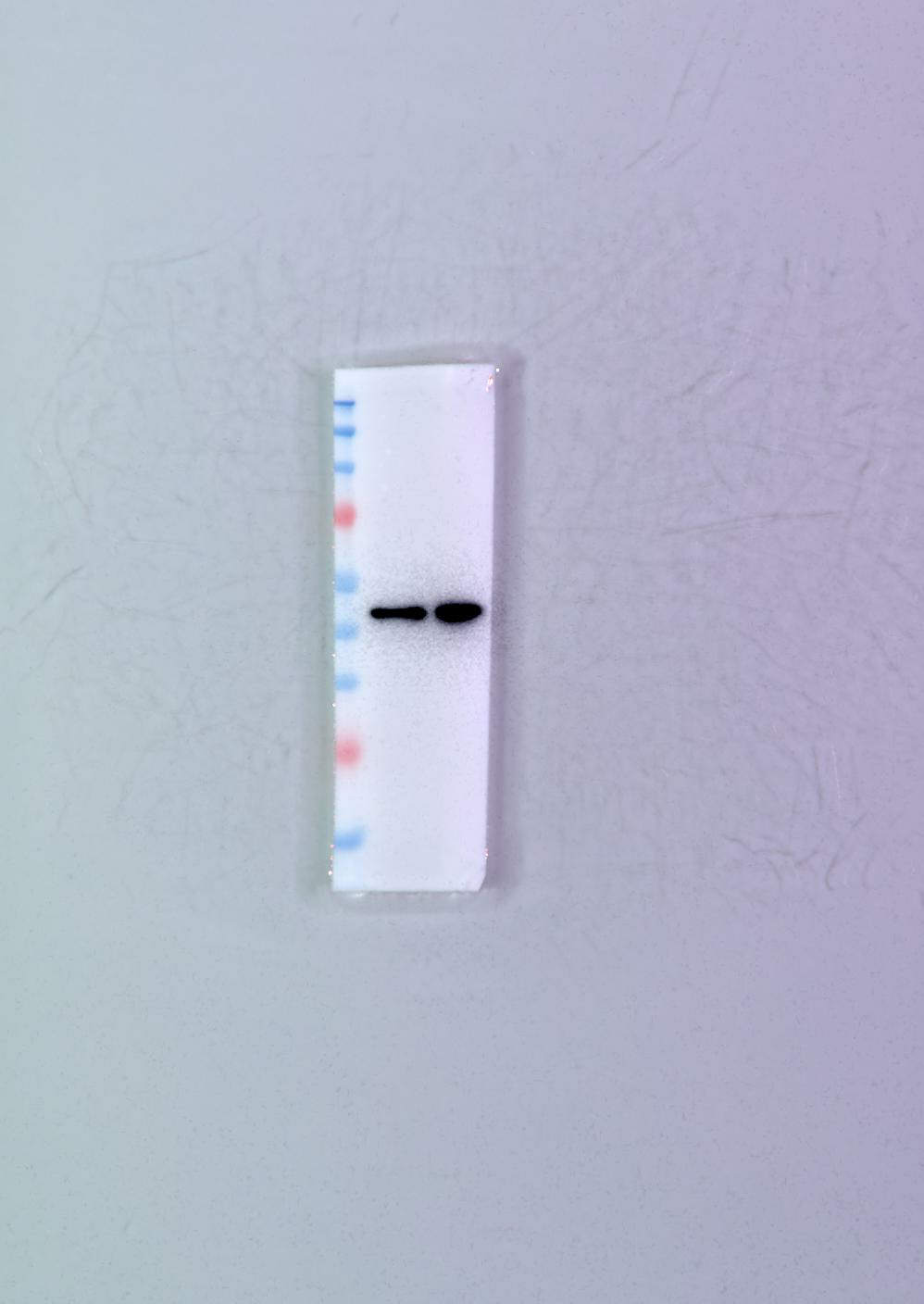

Supplement: Supplementary Image 1 — β-Actin internal control. [file Image_1.tif]

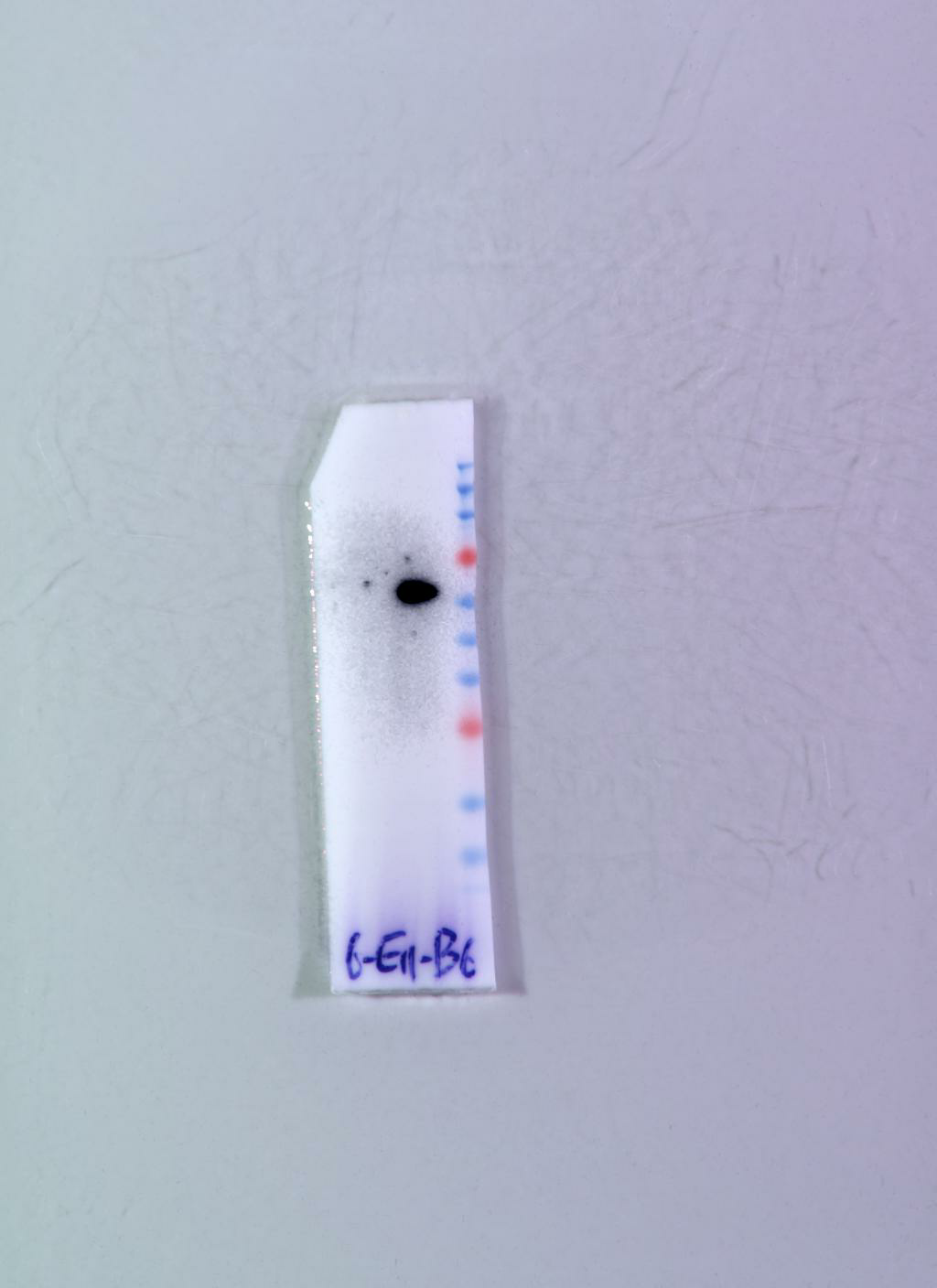

Supplement: Supplementary Image 2 — Penton base protein detection. [file Image_2.tif]
