# Supplementary material for: Isolation and characterization of canine adenovirus type 2 (CAV-HN45) and its selective infection of human cervical cancer cells with preliminary oncolytic potential
Source: Front Vet Sci. 2025 Oct 28;12:1692395. doi: 10.3389/fvets.2025.1692395 (PMC12604354; doi:10.3389/fvets.2025.1692395)
Supplement: Supplementary file 3 [file Table_1.docx]

**Table 1. Sequences of the primers utilized in this study.**

| **Primers** | **Sequences (5’-3’)** |
| --- | --- |
| HA1 | 5’-CGCGCTGAACATTACTACCTTGTC-3’ |
| HA2 | 5’-CCTAGAGCACTTCGTGTCCGCTT-3’ |
| Penton Base-F | 5’-ATGGAGTTTTCGTCGTCTCCTC-3’ |
| Penton Base-R | 5’-CTAGAAGGTTTTACTGGACAGC-3’ |
| Fiber-F | 5’-TTGGTACTTCCACTTGTGCG-3’ |
| Fiber-R | 5’-TAACTTTTCCTGAAGGCGGC-3’ |
| Hexon-F | 5’-TGAGAAGATGGCAACCCCGTCGATG-3’ |
| Hexon-R | 5’-TATTAGCTTAGGTGGTGGCGTTGCC-3’ |
