# Supplementary material for: Isolation and characterization of canine adenovirus type 2 (CAV-HN45) and its selective infection of human cervical cancer cells with preliminary oncolytic potential
Source: Front Vet Sci. 2025 Oct 28;12:1692395. doi: 10.3389/fvets.2025.1692395 (PMC12604354; doi:10.3389/fvets.2025.1692395)
Supplement: Supplementary file 4 [file Table_2.docx]

**Table 2. Relative information of the CAV-HN45 in this study.**

| **Gene** | **Accession number** | **Size CAV-2 (Nucleotide)** | **Size CAV-2 (Amino Acid)** |
| --- | --- | --- | --- |
| Fiber | OP618113 | 1629 bp | 57 kDa |
| Penton Base | OP618114 | 1434 bp | 53.6 kDa |
| Hexon | OP618115 | 2718 bp | 101.2 kDa |
| E3 | OP618116 | 1031 bp | - |
