# Supplementary material for: Isolation and characterization of canine adenovirus type 2 (CAV-HN45) and its selective infection of human cervical cancer cells with preliminary oncolytic potential
Source: Front Vet Sci. 2025 Oct 28;12:1692395. doi: 10.3389/fvets.2025.1692395 (PMC12604354; doi:10.3389/fvets.2025.1692395)
Supplement: Supplementary file 5 [file Table_3.docx]

**Table 3. Summary of human and animal cell lines and their susceptibility to CAV-HN45 infection as determined by CPE and IFA.**

| **Cell line information** | | | **Results of in vitro infection with CAV-HN45** | |
| --- | --- | --- | --- | --- |
| **Species and/or tissue origin** | **Name** | **ATCC no.** | **IFA** | **CPE** |
| Swine |  |  |  |  |
| Kidney | LLC-PK1 | CL-101 | - | - |
|  | PK15 | CCL-33 | + | - |
| Alveolus | 3D4/21 | CRL-2845 | + | - |
| Testis | ST | CRL-1746 | ++ | - |
| Human |  |  |  |  |
| Kidney | 293T |  |  |  |
| Lung carcinoma | A549 | CCL-185EMT | - | - |
| Cervix adenocarcinoma | HeLa | CCL-2 | +++ | ++++ |
| Monkey |  |  |  |  |
| African green monkey kidney | Vero | CRL-1586 | - | - |
| Kidney | Marc-145 | N/A | - | - |
| Mouse |  |  |  |  |
| Embryo fibroblasts | NIH/3T3 | CRL-1658 | - | - |
| Cat |  |  |  |  |
| Kidney | F81 | N/A | - | - |
| Canine |  |  |  |  |
| Kidney | MDCK | CCL-34 | ++++ | ++++ |

a. Degree of infection as determined by IFA or CPE (-, no infection or obvious lesion [≤ 1%]; +, ≤ 25%; ++, ≤ 50%; +++, ≤ 75%; ++++, ≤ 100%).

b. N/A, Not available.
